# New Naphtho-γ-Pyrones Isolated from Marine-Derived Fungus *Penicillium* sp. HK1-22 and Their Antimicrobial Activities

**DOI:** 10.3390/md17060322

**Published:** 2019-05-31

**Authors:** Yao-Yao Zheng, Zhao-Yang Liang, Nan-Xing Shen, Wen-Long Liu, Xiao-Jian Zhou, Xiu-Mei Fu, Min Chen, Chang-Yun Wang

**Affiliations:** 1 Marine Science & Technology Institute, College of Environmental Science & Engineering, Yangzhou University, 196#, Huayang West Street, Yangzhou City 225127, China; zhengyaoyao1210@163.com (Y.-Y.Z.); zhaoyangliangz@163.com (Z.-Y.L.); shennanxing@126.com (N.-X.S.); zhouxiaojian@yzu.edu.cn (X.-J.Z.); 2 Key Laboratory of Marine Drugs, the Ministry of Education of China, School of Medicine and Pharmacy, Ocean University of China, Qingdao 266003, China; xiumei@ouc.edu.cn; 3 Laboratory for Marine Drugs and Bioproducts, Qingdao National Laboratory for Marine Science and Technology, Qingdao 266237, China; 4 College of Chemistry and Chemical Engineering, Yangzhou University, 180#, Siwangting Road, Yangzhou City 225002, China; liuwl@yzu.edu.cn; 5 Institute of Evolution & Marine Biodiversity, Ocean University of China, Qingdao 266003, China

**Keywords:** marine-derived fungus, *Penicillium* sp. HK1-22, naphtho-γ-pyrones, antibacterial activity, antifungal activity

## Abstract

Three novel monomeric naphtho-γ-pyrones, peninaphones A–C (compounds **1**–**3**), along with two known bis-naphtho-γ-pyrones (compounds **4** and **5**) were isolated from mangrove rhizosphere soil-derived fungus *Penicillium* sp. HK1-22. The absolute configurations of compounds **1** and **2** were determined by electronic circular dichroism (ECD) spectra, and the structure of compound **3** was confirmed by single-crystal X-ray diffraction analysis. Compounds **4** and **5** are a pair of hindered rotation isomers. A hypothetical biosynthetic pathway for the isolated monomeric and dimeric naphtho-γ-pyrones is also discussed in this study. Compounds **1**–**3** showed antibacterial activity against *Staphylococcus aureus* (ATCC 43300, 33591, 29213, and 25923) with minimum inhibitory concentration (MIC) values in the range of 12.5–50 μg/mL. Compound **3** exhibited significant activity against the rice sheath blight pathogen *Rhizoctonia solani*.

## 1. Introduction

Natural marine products have been recognized as a valuable and excellent source of structurally novel pharmaceuticals. Marine-derived microorganisms, especially mangrove-associated microorganisms, have developed distinctive metabolic mechanisms on account of the unique properties of the marine environment and their specific functions in the ecosystem, providing a lot of structurally diverse secondary metabolites with a wide variety of biological activities. In recent decades, mangrove-derived fungi have become one of the research hotspots in the area of natural products and marine drugs [1,2,3,4,5,6], with a steady and continued growth in the number of new metabolites, from 108 in 2014 and 126 in 2015 to 142 in 2016 [7].

Naphtho-γ-pyrones, isolated from multiple fungal genera, including *Chaetomium*, *Guanomyces*, *Aspergillus*, *Ustilaginoidea,* and *Penicillium* [8,9,10,11,12,13], are a class with a skeletal structural formula composed of a tricyclic system incorporating naphthalene and fused γ-pyrone rings. Structurally, they are divided into linear and angular shapes according to whether the naphthalene and pyrone rings are in a straight line [14]. Furthermore, they usually occur as dimeric forms, and only a small quantity of monomeric compounds has been reported [9,15,16]. In terms of bioactivities, naphtho-γ-pyrones have been reported for cytotoxicity against human epidermoid carcinoma cells [17], inhibitory effects on HIV-1 integrase [18], triacylglycerol synthesis in mammalian CHO-K1 cells [13], and mouse spleen cell proliferation [19], as well as phytotoxic [11], antitubercular [16], and antimicrobial activities [19]. 

**Figure 1:**
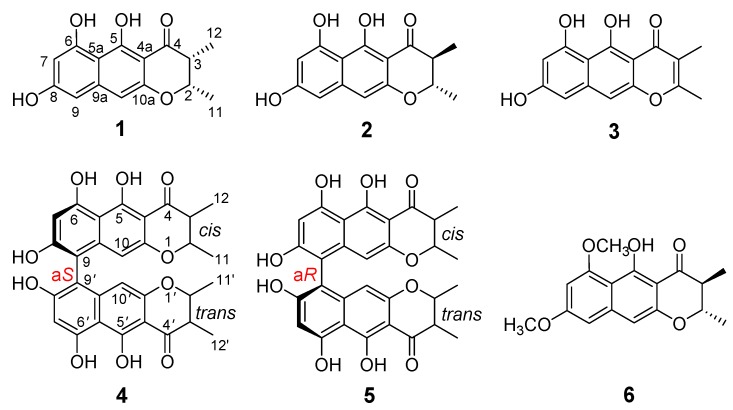
Structures of compounds **1**–**6**.

In our ongoing research on bioactive secondary metabolites produced by mangrove-derived fungi [20,21,22], a strain of *Penicillium* sp. HK1-22 isolated from mangrove rhizosphere soil attracted our attention, because its culture extract showed antibacterial activity against a panel of pathogenic bacteria. Chemical investigation of this fungus cultivated with potato dextrose broth led to the isolation of three new monomeric naphtho-γ-pyrones, peninaphones A–C (Figure 1, compounds **1**–**3**), along with two known bis-naphtho-γ-pyrones (Figure 1, compounds **4** and **5**). In this study, the isolation, structure elucidation, and biological activities of compounds **1**–**5** are described. A hypothetical biosynthetic pathway for the isolated naphtho-γ-pyrones (compounds **1**–**5**) is also discussed.

## 2. Results and Discussion

**Figure 2:**
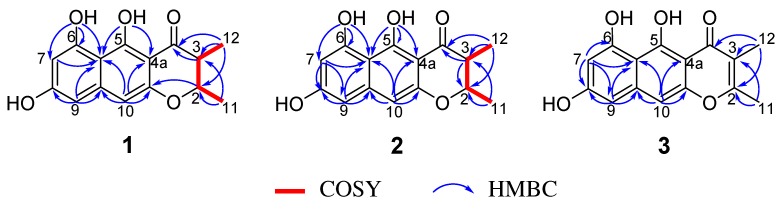
COSY and key HMBC correlations for compounds **1**–**3**.

Peninaphone A (compound **1**) was isolated as a yellow powder and exhibited the molecular formula of C_15_H_14_O_5_ (nine degrees of unsaturation) based on high resolution electrospray ionization mass spectroscopy (HRESIMS) ([M + H]^+^ 275.0857 (calculated for C_15_H_15_O_5_ 275.0860)) and NMR spectroscopic data. The IR spectrum of compound **1** revealed the presence of hydroxyl (3379 cm^−1^) and carbonyl (1642 cm^−1^). Its ^1^H NMR (Table 1) spectrum revealed two methyl doublet signals at δ_H_ 1.20 (3 H, d, *J* = 6.6 Hz) and 1.38 (3 H, d, *J* = 6.6 Hz), two methine groups at δ_H_ 2.82 (1 H, dq, *J* = 3.0, 6.6 Hz) and 4.67 (1 H, dq, *J* = 3.0, 6.6 Hz), and three aromatic signals at δ_H_ 6.28 (1 H, brs), 6.46 (1 H, s), and 6.52 (1 H, brs), as well as three hydroxyl protons at δ_H_ 9.15 (1 H, s), 9.48 (1 H, s), and 15.59 (1 H, s). The ^13^C NMR (Table 1) and distortionless enhancement by polarization transfer (DEPT) spectrum displayed 15 carbon resonance signals, which were assigned to one carbonyl (δ_C_ 203.2), 10 aromatic carbon atoms, two methines, and two methyl groups. These fragments accounted for six of the nine degrees of unsaturation, requiring three additional rings to be present in compound **1**. The above NMR spectroscopic data showed that compound **1** was very similar to (2*S*,3*S*)-5-hydroxy-6,8-dimethoxy-2,3-dimethyl-4*H*-2,3- dihydronaphtho[2,3-b]-pyran-4-one (Figure 1, compound **6**), a naphtho-γ-pyrone equipped with a linear tricyclic system isolated from coprophilous fungus *Guanomyces polythrix* [9]. The obvious difference in the ^1^H NMR spectrum was the disappearance of two oxygenated methyl groups with the concomitant presence of an additional two hydroxyl signals, suggestive of three hydroxyl groups in compound **1**. In the heteronuclear multiple bond correlation (HMBC) spectra (Figure 2), correlations from OH-6 (δ_H_ 9.48) to C-5a (δ_C_ 105.0), C-6 (δ_C_ 160.8), and C-7 (δ_C_ 100.7) and from OH-5 to C-4a (δ_C_ 101.8) and C-5a (δ_C_ 105.0) enabled two hydroxyl groups to be placed at C-6 and C-5, respectively. The remaining hydroxyl group could be deduced at C-8 according to a comparison of the ^13^C NMR chemical shift of C-8 (δ_C_ 162.9 in compound **1** vs. δ_C_ 162.3 in compound **6**), although the HMBC connection from OH-8 (δ_H_ 9.15) to C-8 (δ_C_ 162.9) could not be observed for compound **1**. Detailed assignments for proton and carbon signals (Table 1) for compound **1** were unambiguously accomplished by analysis of its one-dimensional (1D) and two-dimensional (2D) NMR data.

The relative configuration of compound **1** was determined by the analysis of its coupling constant. The small coupling constant (*J* = 3.0 Hz) between the two methine protons (H-2 and H-3) in the dihydro-γ-pyrone ring suggested the *cis* orientation of 2-H and 3-H. The absolute configuration of compound **1** was established by its electronic circular dichroism (ECD) spectrum (Figure S7) according to previous reports [9,23]. The ECD spectrum of compound **1** displayed negative Cotton effect around 314 nm, suggesting the 2*S* configuration for compound **1**. Automatically, the configuration at C-3 was assigned as *R* in compound **1** due to the established *cis* relationship of the methyl groups. Therefore, the absolute configuration of compound **1** was assigned as 2*S*,3*R*.

Peninaphone B (compound **2**) exhibited the same molecular formula as compound **1** according to its HRESIMS data ([M + H] ^+^ 275.0907 (calculated for C_15_H_15_O_5_ 275.0914)). The 1D and 2D NMR data of compound **2** (Table 1) were almost identical to those of compound **1**, indicating that these two compounds were isomers sharing the same planar structure. It is worth noting that their NMR data changed slightly in the γ-pyrone part. Firstly, differences were found for the chemical shifts of the methines H-2/C-2 (δ_H_/δ_C_ 4.67/76.4 in compound **1** vs. 4.29/79.1 in compound **2**) and H-3/C-3 (δ_H_/δ_C_ 2.82/44.9 in compound **1** vs. 2.80/46.7 in compound **2**) and the methyls CH_3_-11 (δ_H_/δ_C_ 1.38/16.6 in compound **1** vs. 1.50/19.9 in compound **2**) and CH_3_-12 (δ_H_/δ_C_ 1.20/9.8 in compound **1** vs. 1.25/10.3 in compound **2**). Secondly, the coupling constant values of H-2 and H-3 in compound **2** were significantly changed (*J* = 3.0 Hz in compound **1** vs. *J* = 10.8 Hz in compound **2**), clearly indicating that H-2 and H-3 in compound **2** were in a *trans* relationship. The ECD spectrum of compound **2** displayed negative Cotton effect around 314 nm (Figure S14), suggesting the same 2*S* absolute configuration for compound **2**. Combined with the *trans* configuration of H-2 and H-3, the absolute configuration of compound **2** was established as 2*S*,3*S*. 

**Figure 3:**
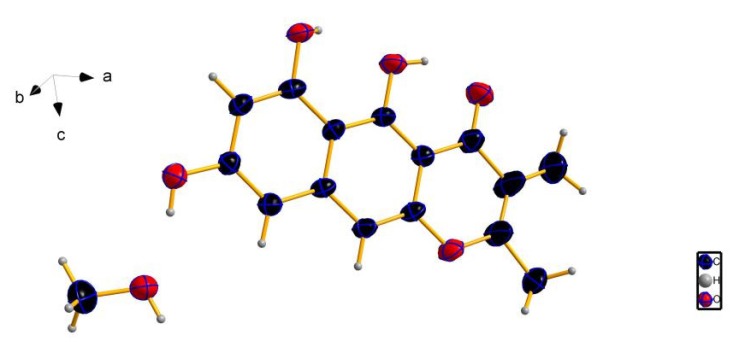
Oak Ridge thermal ellipsoid plot (ORTEP)-like view of the molecular structure of compound **3**.

Peninaphone C (compound **3**) was also obtained as a yellow powder, giving the molecular formula C_15_H_12_O_5_ with 10 degrees of unsaturation by the analysis of its HRESIMS, revealing two fewer H than compounds **1** and **2**. The presence of two nonprotonated vinylic signals (δc 165.2 and 111.8) in the ^13^C NMR spectrum of compound **3** suggested that it was an unsaturated analogue of compound **1**. Comprehensive analysis of 1D and 2D NMR data of compounds **1** and **3** revealed that the CH–CH moiety of compound **1** was replaced by a double bond at C-2(3) in compound **3**. Furthermore, by slow crystallization from CH_3_OH, single crystals of compound **3** were obtained, which were suitable for X-ray analysis. Subsequently, by X-ray crystallography, the structure of compound **3** was unequivocally determined, and its Oak Ridge thermal ellipsoid plot (ORTEP)-like view is shown in Figure 3.

Compounds **4** and **5** were elucidated as two isomers of bis (naphthodihydropyran-4-one) with the same planar structure. The ^1^H NMR data showed that they were asymmetrical dimers having one *cis*-2,3-dimethyl group and one *trans*-2,3-dimethyl group in dihydro-γ-pyrone rings, respectively. The ECD spectra of compounds **4** and **5** (Figure S20) suggested that the structural difference between them comes from the 9/9*’* chiral axis (a*S* or a*R*) of the two monomers. The ECD curve of compound **4** showed positive Cotton effect at 295 nm and negative Cotton effect at 265 nm, indicating that compound **4** possesses an a*S* 9,9*’*-axial chirality [24,25]. On the contrary, compound **5** displayed a mirror image ECD spectra relative to compound **4**; therefore, an a*R* 9,9*’*-axial stereochemistry was assigned to compound **5**. Therefore, compounds **4** and **5** were identified as a pair of hindered rotation isomers isochaetochromins B_1_ and B_2_, respectively, by the comparison of their ECD, NMR, and electrospray ionization-mass spectrometry (ESIMS) data with those reported in the previous literature [13]. 

A research of the literature revealed that, in general, the mono- and bis-naphtho-γ-pyrones were rarely obtained in the same strain simultaneously. For example, 10 new monomeric naphtho-γ-pyrones were isolated from *Guanomyces polythrix* [9,15], but no bis-naphtho-γ-pyrones appeared. Again, Zhou’s research group obtained 15 new bis-naphtho-γ-pyrones, ustilaginoidins K–T, from *Ustilaginoidea virens* (*Villosiclava virens*) [11,12], but no monomeric naphtho-γ-pyrones were isolated. However, in the present study, three monomeric (compounds **1**–**3**) and two related bis-naphtho-γ-pyrones (compounds **4** and **5**) were isolated from strain HK1-22 at the same time. 

**Scheme 1:**
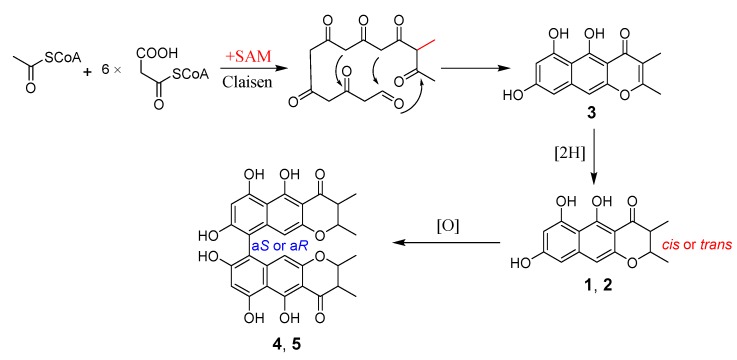
Proposed biosynthetic pathway of compounds **1**–**5**. SAM: *S*-adenosylmethionine.

A plausible biosynthetic pathway for the isolated naphtho-γ-pyrones (compounds **1**–**5**) was proposed (Scheme 1), where the monomeric metabolites were produced via the polyketide pathway and the dimers formed through oxidative coupling [11]. The biosynthesis was started from Claisen condensation of one acetyl-CoA starter unit, six malonyl-CoA extender units, and one methyl unit donated by *S*-adenosylmethionine (SAM); then, compound **3** was produced and further reduced to form its dihydro derivatives, compounds **1** and **2**. The oxidative coupling of compounds **1** and **2** yielded the dimeric compounds **4** and **5** with different axial chirality (Scheme 1). In the previous literature [11], the oxidative coupling between monomeric naphtho-γ-pyrone radicals was proposed to yield the dimeric compounds, but no monomer was isolated from the fungal strain. Noteworthy in this paper, the monomeric naphtho-γ-pyrones and related bis-naphtho-γ-pyrones were obtained simultaneously for the first time, which confirmed the rationality of the proposed biosynthetic pathway.

Naphtho-γ-pyrones are a large class of fungal secondary metabolites possessing a wide range of biological activities [11,12,13,14,15,16,17,18,19]. In this study, the antibacterial effects of the isolated compounds (**1**–**5**) are tested against seven bacterial strains, including gram-positive *Staphylococcus aureus* (ATCC 43300, 33591, 29213, and 25923), *Enterococcus faecalis* (ATCC 51299), *Enterococcus faecium* (ATCC 35667), and gram-negative *Escherichia coli* (ATCC 25922). The results (Table 2) indicate that monomeric compounds **1**–**3** showed similar antibacterial activities against the four strains of *S. aureus* with minimum inhibitory concentration (MIC) values in the range of 12.5–50 μg/mL. It is worth noting that compounds **1**–**3** exhibited moderate antibacterial activities against methicillin-resistant *S. aureus* (ATCC 43300 and ATCC 33591) with the MIC values of 12.5 and 25 μg/mL, respectively. Neither of the two dimers (compounds **4** or **5**) showed activity against the tested bacterial strains. Comparing the antibacterial activities of the monomeric compounds (**1**–**3**) and the two dimers (compounds **4** and **5**), the results suggest that the dimeric forms decrease the antibacterial activity.

Phytopathogens can cause significant crop failure and financial loss; therefore, the screening of microbial metabolites for agricultural fungicide has become a hot topic in recent years [26,27,28]. In an attempt to discover a potential new fungicide, the antifungal activities in vitro of compound **3** were screened by the mycelium linear growth rate method against four crop pathogenic fungi, including *Rhizoctonia solani*, *Rhizoctonia cerealis*, *Gaeumannomyces graminis*, and *Alternaria alternata* at 50, 10, and 2 μg/mL. The results showed that compound **3** exhibited significant antifungal activities against the four tested crop pathogens (Table 3), especially against the rice sheath blight pathogen *R. solani*.

Meanwhile, compounds **1**–**3** were screened for their cytotoxic activities against the human hepatocellular carcinoma cell line SMMC-7721. However, none of these monomeric compounds were proved to be active.

## 3. Materials and Methods

### 3.1. General Experimental Procedures

IR spectra were recorded on a Cary 610/670 spectrometer (Varian, Salt Lake City, UT, USA) using KBr pellets. UV spectra were measured on a Beckman DU 640 spectrophotometer (Beckman Coulter, Kraemer Boulevard Brea, CA, USA). Optical rotations were acquired on an Anton Paar MCP300 automatic polarimeter (Anton Paar, Graz, Austria). 1D and 2D NMR spectra were recorded on an Avance 600 NMR spectrometer (Bruker, Karlsruhe, Germany)(600 MHz for ^1^H and 150 MHz for ^13^C), using TMS (tetramethylsilane) as an internal standard. HRESIMS spectra were obtained from a maXis spectrometer (Bruker, Karlsruhe, Germany). Single-crystal data were determined on a Bruker D8 Quest diffractometer (Mo Kα radiation) (Bruker, Karlsruhe, Germany). Semi-preparative HPLC was performed on a Hitachi system (Hitachi, Tokyo, Japan) using a semi-preparative C_18_ (Kromasil, 5 μm, 10 × 250 mm) column coupled with a 2400 UV detector. Silica gel (200–300 mesh; Qing Dao Hai Yang Chemical Group Co., Qing Dao, China), Sephadex LH-20 (Amersham Biosciences, Uppsala, Sweden), and octadecylsilyl silica gel (45–60 μm; Unicorn) were used for column chromatography (CC). Precoated silica gel plates (G60, F-254; Yan Tai Zi Fu Chemical Group Co., Yan Tai, China) were used for thin layer chromatography (TLC).

### 3.2. Fungal Material 

*Penicillium* sp. HK1-22 was isolated from mangrove rhizosphere soil, which was collected from the Dongzhaigang mangrove natural reserve in Hainan Island in September 2015. The strain was preserved at the Marine Science & Technology Institute, College of Environmental Science & Engineering, Yangzhou University, Yangzhou, China and identified according to its morphological traits and molecular protocol by amplification and sequencing of the DNA of the internal transcribed spacer (ITS) region of the rRNA gene as described previously [29]. The 593 base pair ITS sequence was submitted to GenBank with the accession number MK790264, and 99% sequence identity resulted with *Penicillium javanicum* CBS 129771 (MH877078.1).

### 3.3. Fermentation, Extraction, and Isolation 

The *Penicillium* sp. HK1-22 strain was statically fermented in 75 × 1 L Erlenmeyer flasks containing 400 mL of potato dextrose broth (20 g of dextrose and 30 g of natural sea salt in 1 L of potato infusion) for 4 weeks at room temperature. The culture (30 L) was filtered to separate the broth from the mycelia. The former was exhaustively extracted three times with an equal volume of EtOAc, while the latter was extracted three times with MeOH. The organic extracts were combined and concentrated in vacuo to afford 51.0 g of the total extract, which was subjected to vacuum liquid chromatography (VLC) on silica gel using solvents in a gradient of increasing polarity—petroleum ether–EtOAc–MeOH—to yield a total of five fractions (Fr.1–Fr.5). Fr.3 was subjected to Sephadex LH-20 CC with mixtures of CHCl_2_–MeOH (1:1, v/v) to obtain subfractions Fr.3-1–Fr.3-3. Fr.3-2 was further purified by semi-preparative HPLC, eluting with MeOH–H_2_O (70:30, v/v) to yield compound **1** (50.0 mg) and compound **2** (40.0 mg). Recrystallization of Fr.3-3 obtained compound **3** (260.0 mg). Fr.5 was repeatedly subjected to silica gel CC, purified by octadecylsilyl (ODS) CC, and, finally, purified by semipreparative HPLC eluting with MeOH–H_2_O (65:35, v/v) to give compound **4** (2.0 mg) and compound **5** (3.0 mg).

Peninaphone A (compound **1**): yellow amorphous powder; [α]^20^_D_ +89 (*c*, 0.05, MeOH); UV (MeOH) λ_max_ (log *ε*) 332 (0.5), 282 (2.5), 232 (1.8) nm; ECD (1.24 mM, MeOH) λ_max_ (Δ*ε*) 339 (−2.35), 325 (+0.04), 313 (−2.10), 296 (+2.73), 280 (−11.67), 247 (+4.51), 231 (−2.07) nm; IR (KBr) *ν*_max_ 3379, 2948, 2843, 1642, 1454, 1361, 1054, 1032 cm^–1^; ^1^H and ^13^C NMR data (Table 1); HRESIMS *m/z* 275.0857 [M + H]^+^ (calculated for C_15_H_15_O_5_, 275.0860).

Peninaphone B (compound **2**): yellow amorphous powder; [α]^20^_D_ +86 (*c*, 0.06, MeOH); UV (MeOH) λ_max_ (log *ε*) 328 (0.4), 280 (2.2), 232 (1.5) nm; ECD (1.24 mM, MeOH) λ_max_ (Δ*ε*) 336 (−1.69), 325 (−0.21), 294 (−11.81), 256 (5.01), 237 (−3.34) nm; IR (KBr) *ν*_max_ 3377, 2948, 2833, 1641, 1455, 1419, 1032 cm^–1^; ^1^H and ^13^C NMR data, see Table 1; HRESIMS *m/z* 275.0907 [M + H]^+^ (calculated for C_15_H_15_O_5_, 275.0914).

Peninaphone C (compound **3**): yellow amorphous powder; UV (MeOH) λ_max_ (log *ε*) 310 (0.05), 278 (0.3), 216 (0.11) nm; IR (KBr) *ν*_max_ 3382, 2948, 2843, 1641, 1454, 1054, 1032 cm^−1^; ^1^H and ^13^C NMR data, see Table 1; HRESIMS *m/z* 273.0747 [M + H]^+^ (calculated for C_15_H_13_O_5_, 273.0757).

### 3.4. X-ray Crystallographic Analysis of Compound ***3***

A colorless crystal of compound **3** was obtained by the slow evaporation of a methanol solution. All crystallographic data were collected at 296 K on a Bruker D8 Quest diffractometer with Mo Kα radiation (λ = 0.71073 Å). The structure was elucidated by direct methods using a SHELXS-97 and refined by means of full-matrix least-squares on F^2^. All non-hydrogen atoms were refined anisotropically. The hydrogen atoms were located by geometrical calculations, and their positions and thermal parameters were determined during the structure refinement. Crystallographic data for compound **3** have been deposited in the Cambridge Crystallographic Data Centre as supplementary publication number CCDC 1909893. 

Crystal data for compound **3**: C_15_H_12_O_5_**·**CH_3_OH, M_r_ = 304.29, monoclinic, *a* = 9.2406(14) Å, *b* = 12.9091(19) Å, *c* = 12.6168(17) Å, α = 90°, β = 91.827(5)°, γ = 90°, *V* = 1504.3(4) Å^3^, space group *P*2**_1_**/c, Z = 4, *Dx* = 1.344 mg/mm^3^, μ(Mo Kα) = 0.103 mm^−1^, and *F*(000) = 640. Crystal dimensions: 0.150 × 0.120 × 0.080 mm^3^. Reflections collected: 19,440. Independent reflections: 2634 (Rint = 0.0522). The final *R*_1_ values were 0.0987, w*R*_2_ = 0.2363 (*I* > 2σ(*I*)), *R* indices for all data *R*_1_ = 0.1201, and w*R*_2_ = 0.2497.

### 3.5. Biological Assays

#### 3.5.1. Antibacterial Assay

The antibacterial assay was carried out as described previously [30].

#### 3.5.2. Anti-Phytopathogenic Assay

The antifungal activities of four crop pathogenic fungi (*R. Solani*, *R. cerealis*, *G. graminis*, and *A. alternata*) were assayed in vitro by the mycelium linear growth rate method according to [31].

#### 3.5.3. Cytotoxic Assay

The cytotoxic activity was evaluated against the human hepatocellular carcinoma cell line SMMC-7721 by the MTT (3-(4,5-dimethyl-2-thiazolyl)-2,5-diphenyl-2-*H*-tetrazolium bromide, thiazolyl blue tetrazolium bromide) method as described in [32]. Cisplatin was used as a positive control.

## 4. Conclusions 

In summary, three new monomeric naphtho-γ-pyrones (compounds **1**–**3**) and two known bis-naphtho-γ-pyrones (compounds **4** and **5**) were isolated from mangrove rhizosphere soil-derived fungus *Penicillium* sp. HK1-22. Compounds **1** and **2** were a pair of monomeric isomers, whereas compounds **4** and **5** were a pair of hindered rotation isomers. Monomeric naphtho-γ-pyrones and related bis-naphtho-γ-pyrones were simultaneously obtained from a fungal strain for the first time. Compounds **1**–**3** showed moderate antibacterial activities against four strains of *S. aureus* with MIC values in the range of 12.5–50 μg/mL. Compound **3** was also found to exhibit significant antifungal activities against the four tested crop pathogens.

## Figures and Tables

**Table 1:** NMR spectroscopic data (600 MHz, acetone-*d*_6_) for compounds **1**–**3**.

Position	1		2		3	
	δ_C_ Type	δ_H_ (Mult. *J* in Hz)	δ_C_ Type	δ_H_ (Mult. *J* in Hz)	δ_C_ Type	δ_H_ (Mult. *J* in Hz)
2	76.4, CH	4.67 (dq, 3.0, 6.6)	79.1, CH	4.29 (dq, 10.8, 6.0)	165.2, C	
3	44.9, CH	2.82 (dq, 3.0, 6.6)	46.7, CH	2.80 (dq, 10.8, 6.6)	111.8, C	
4	203.2, C		201.6, C		182.6, C	
4a	101.8, C		102.3, C		101.3, C	
5	166.3, C		165.6, C		162.0, C	
5a	105.0, C		105.0, C		105.2, C	
6	160.8, C		160.8, C		158.3, C	
7	100.7, CH	6.28 (brs)	100.7, CH	6.27 (brs)	100.3, CH	6.31 (d, 1.8)
8	162.9, C		162.9, C		160.6, C	
9	102.3, CH	6.52 (brs)	102.3, CH	6.51 (brs)	100.4, CH	6.56 (d, 1.8)
9a	143.6, C		143.6, C		140.3, C	
10	101.5, CH	6.46 (s)	101.4, CH	6.45 (s)	99.2, CH	6.95 (s)
10a	155.8, C		156.1, C		151.4, C	
11	16.6, CH_3_	1.38 (d, 6.6)	19.9, CH_3_	1.50 (d, 6.0)	18.6, CH_3_	2.38 (s)
12	9.8, CH_3_	1.20 (d, 6.6)	10.3, CH_3_	1.25 (d, 6.6)	8.6, CH_3_	1.92 (s)
OH-5		15.59 (s)		15.62 (s)		16.05 (s)
OH-6		9.48 (s)		9.46 (s)		10.18 (s)
OH-8		9.15 (s)		9.13 (s)		9.82 (s)

**Table 2:** Antibacterial activities of compounds **1**–**5** ^a^.

Pathogenic Bacteria	Antibiotic Resistant (–)/Susceptible (+)	MIC (μg/mL)							
		1	2	3	4	5	O^b^	V^c^	C^d^
*Staphylococcus aureus* ATCC 43300	methicillin (–)	12.5	12.5	12.5	>50	>50	>50	1.56	–
*Staphylococcus aureus* ATCC 33591	methicillin (–)	25	25	25	>50	>50	>50	0.39	–
*Staphylococcus aureus* ATCC 25923	methicillin (+)	50	50	50	>50	>50	0.39	1.56	–
*Staphylococcus aureus* ATCC 29213	methicillin (+)	25	25	25	>50	>50	0.39	0.78	–
*Enterococcus faecalis* ATCC 51299	vancomycin (–)	>50	>50	>50	>50	>50	>50	>50	–
*Enterococcus faecium* ATCC 35667	vancomycin (+)	>50	>50	>50	>50	>50	>50	0.78	–
*Escherichia coli* ATCC 25922	–	>50	>50	>50	>50	>50	>50	>50	1.56

^a^ Data are expressed in minimum inhibitory concentration (MIC) values: ^b^ O, Oxacillin sodium; ^c^ V, Vancomycin; and ^d^ C, Ciprofloxacin.

**Table 3:** Antifungal activities of compound **3**.

Pathogenic Fungus	Average Inhibition Rate (%)		
	50 μg/mL	10 μg/mL	2 μg/mL
*Rhizoctonia solani*	100	50.2	12
*Rhizoctonia cerealis*	100	30.4	0
*Gaeumannomyces graminis*	98.3	23.1	0
*Alternaria alternata*	80.3	25.9	0

## Supplementary Materials

The following are available online at https://www.mdpi.com/1660-3397/17/6/322/s1, Figures S1–S20: The ECD spectra of compounds **1**, **2**, **4**, and **5**; HRESIMS; and 1D and 2D NMR spectra of compounds **1**–**3**.
