# Supplementary material for: New Naphtho-γ-Pyrones Isolated from Marine-Derived Fungus Penicillium sp. HK1-22 and Their Antimicrobial Activities
Source: Mar Drugs. 2019 May 31;17(6):322. doi: 10.3390/md17060322 (PMC6627118; doi:10.3390/md17060322)
Supplement: Supplementary file 1 [file marinedrugs-17-00322-s001.pdf]

Supplementary Materials

New Naphtho-γ-Pyrones Isolated from Marine-Derived Fungus *Penicillium* sp. HK1-22 and Their Antimicrobial Activities

Yao-Yao Zheng [1,2,3,†], Zhao-Yang Liang [1,2,3,†], Nan-Xing Shen [1], Wen-Long Liu [4], Xiao-Jian Zhou [1], Xiu-Mei Fu [2,3], Min Chen [1,*] and Chang-Yun Wang [2,3,5,*]

[1] Marine Science & Technology Institute, College of Environmental Science & Engineering, Yangzhou University, 196#, Huayang West Street, Yangzhou City 225127, Jiangsu Province, People's Republic of China; zhengyaoyao1210@163.com (Y.-Y.Z.); zhaoyangliangz@163.com (Z.-Y.L.); shennanxing@126.com (N.-X.S.); zhouxiaojian@yzu.edu.cn (X.-J.Z.)

[2] Key Laboratory of Marine Drugs, the Ministry of Education of China, School of Medicine and Pharmacy, Ocean University of China, Qingdao 266003, People's Republic of China; xiumei@ouc.edu.cn (X.-M.F.)

[3] Laboratory for Marine Drugs and Bioproducts, Qingdao National Laboratory for Marine Science and Technology, Qingdao 266237, People's Republic of China

[4] College of Chemistry and Chemical Engineering, Yangzhou University, 180#, Siwangting Road, Yangzhou City, 225002, Jiangsu Province, People's Republic of China; liuwl@yzu.edu.cn (W.-L.L.)

[5] Institute of Evolution & Marine Biodiversity, Ocean University of China, Qingdao 266003, People's Republic of China

* Correspondence: dieying0719@163.com (M.C.); changyun@ouc.edu.cn (C.-Y.W.); Tel.: +86-514-8979-5882 (M.C.); +86-532-8203-1536 (C.-Y.W.)

† These authors contributed equally to this work.

List of Supporting Information

Figure S1. ^{1}H NMR spectrum of compound **1** in acetone-d_6 (600 MHz).

Figure S2. ^{13}C NMR spectrum of compound **1** in acetone-d_6 (150 MHz).

Figure S3. ^{1}H-^{1}H COSY spectrum of compound **1** in acetone-d_6 (600 MHz).

Figure S4. HSQC spectrum of compound **1** in acetone-d_6 (600 MHz).

Figure S5. HMBC spectrum of compound **1** in acetone-d_6 (600 MHz).

Figure S6. HRESIMS spectrum (positive ion mode) of compound **1**.

Figure S7. ECD spectrum of compound **1**.

Figure S8. ^{1}H NMR spectrum of compound **2** in acetone-d_6 (600 MHz).

Figure S9. ^{13}C NMR spectrum of compound **2** in acetone-d_6 (150 MHz).

Figure S10. ^{1}H-^{1}H COSY spectrum of compound **2** in acetone-d_6 (600 MHz).

Figure S11. HSQC spectrum of compound **2** in acetone-d_6 (600 MHz).

Figure S12. HMBC spectrum of compound **2** in acetone-d_6 (600 MHz).

Figure S13. HRESIMS spectrum (positive ion mode) of compound **2**.

Figure S14. ECD spectrum of compound **2**.

Figure S15. ^{1}H NMR spectrum of compound **3** in acetone-d_6 (600 MHz).

Figure S16. ^{13}C NMR spectrum of compound **3** in acetone-d_6 (150 MHz).

Figure S17. HSQC spectrum of compound **3** in acetone-d_6 (600 MHz).

Figure S18. HMBC spectrum of compound **3** in acetone-d_6 (600 MHz).

Figure S19. HRESIMS spectrum (positive ion mode) of compound **3**.

Figure S20. ECD spectrum of compounds **4** and **5**.

Figure S21. HPLC chromatogram of the crude extract from the fungus *Penicillium* sp. HK1-22.

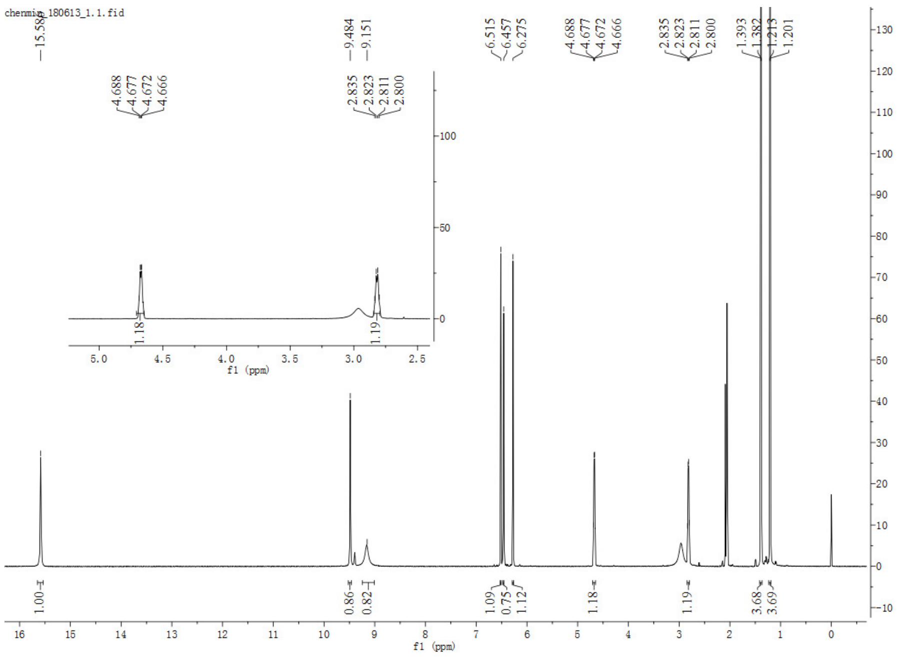

Figure S1. ^{1}H NMR spectrum of compound **1** in acetone-d_6 (600 MHz).

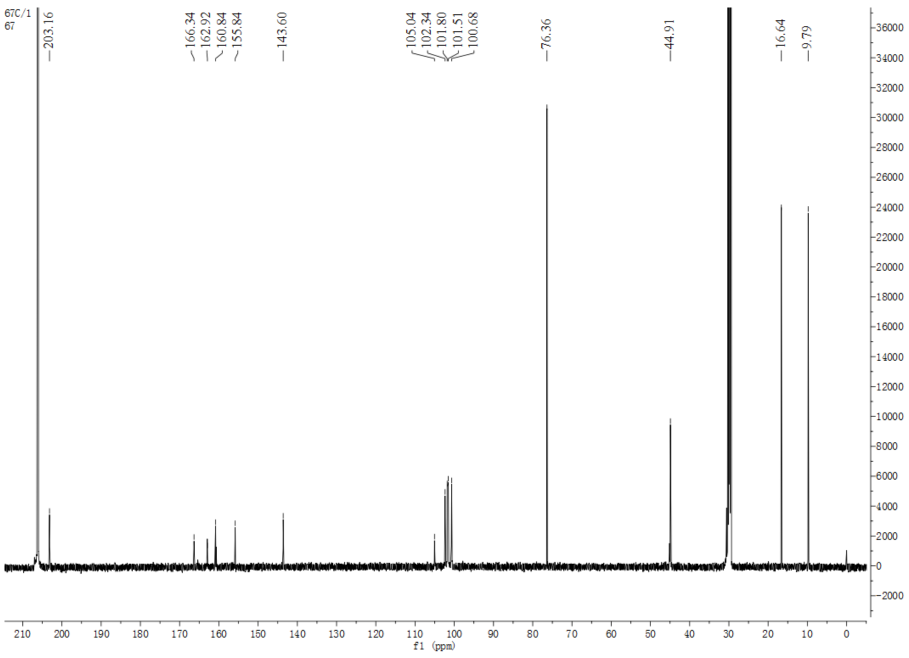

Figure S2. ^{13}C NMR spectrum of compound **1** in acetone-d_6 (150 MHz).

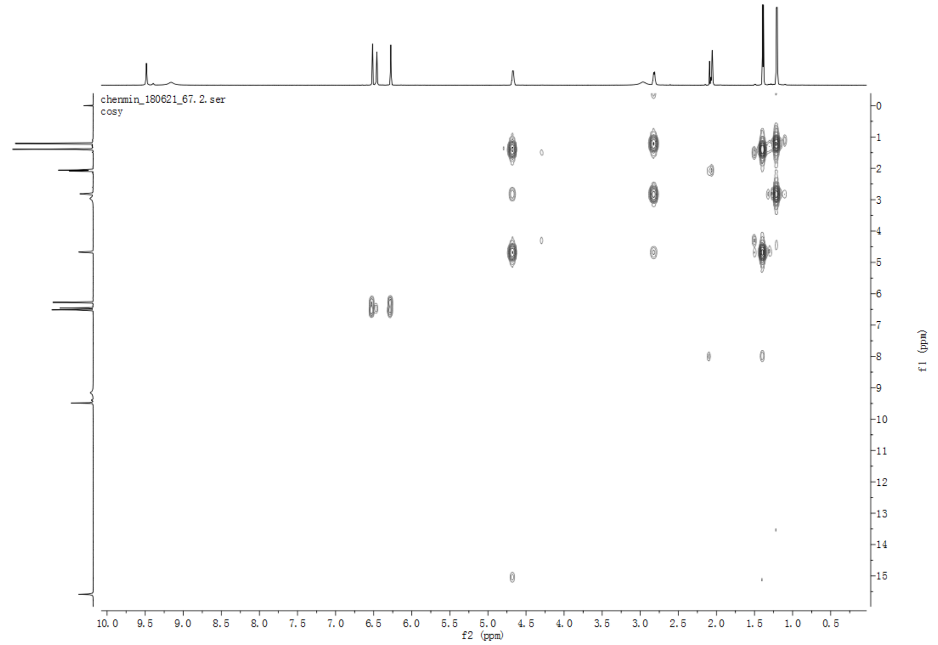

Figure S3. ^{1}H-^{1}H COSY spectrum of compound **1** in acetone-*d*$_6$ (600 MHz).

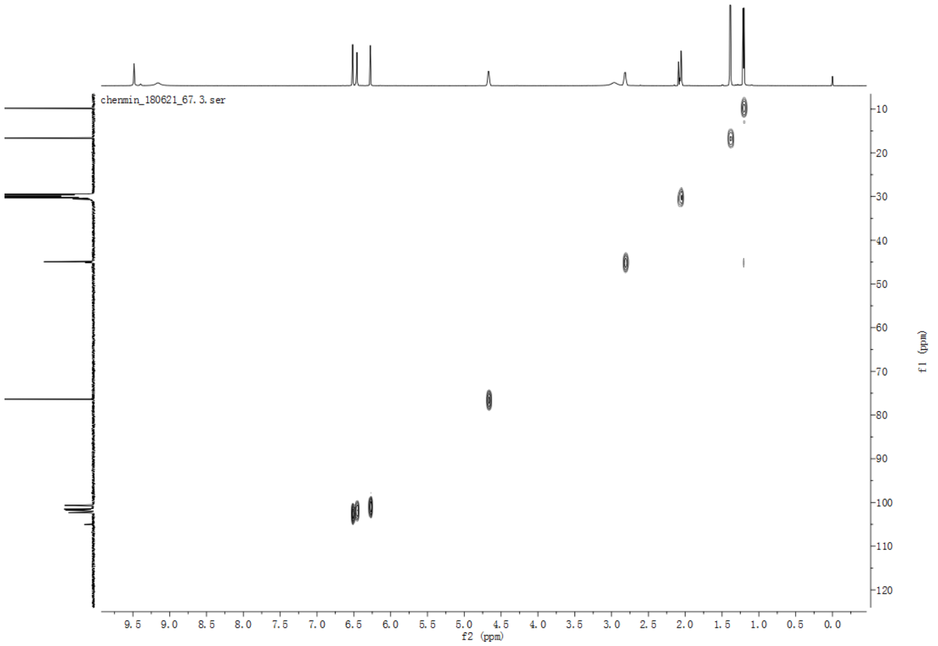

Figure S4. HSQC spectrum of compound **1** in acetone-*d*$_6$ (600 MHz).

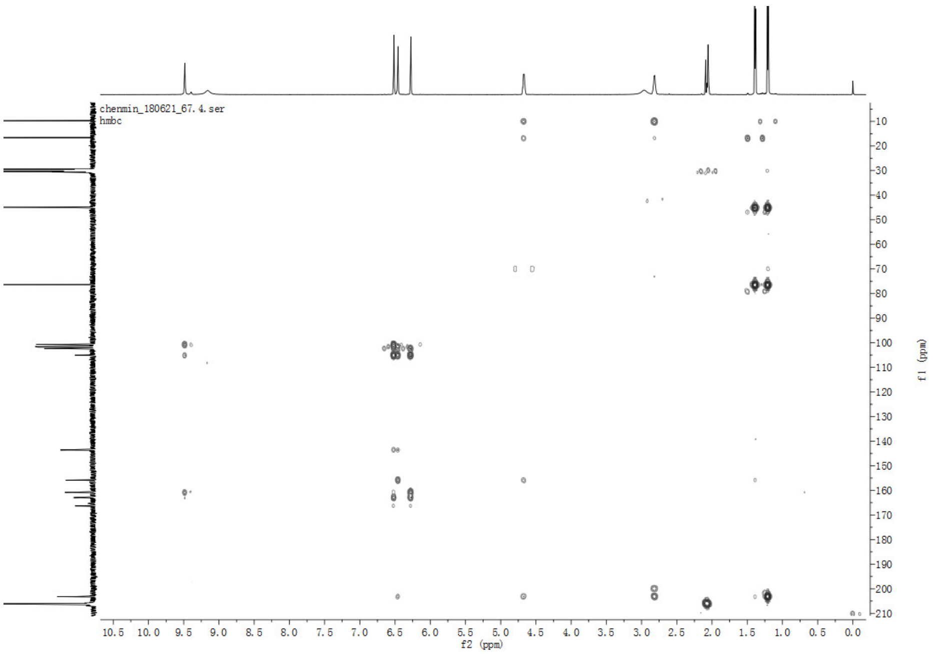

Figure S5. HMBC spectrum of compound **1** in acetone-d_6 (600 MHz).

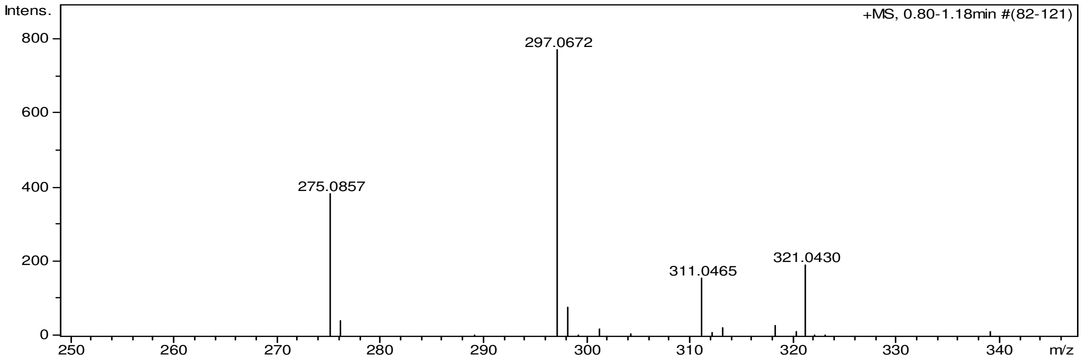

HRESIMS *m/z* 275.0857 (calcd for $C_{15}H_{15}O_5$, 275.0860),

297.0733 (calcd for $C_{15}H_{14}NaO_5$, 275.0860)

Figure S6. HRESIMS spectrum (positive ion mode) of compound **1**.

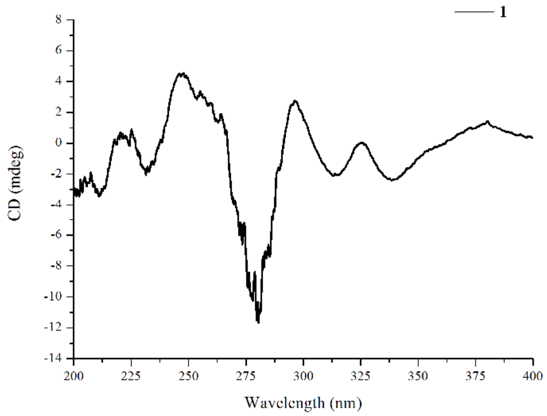

Figure S7. ECD spectrum of compound **1**.

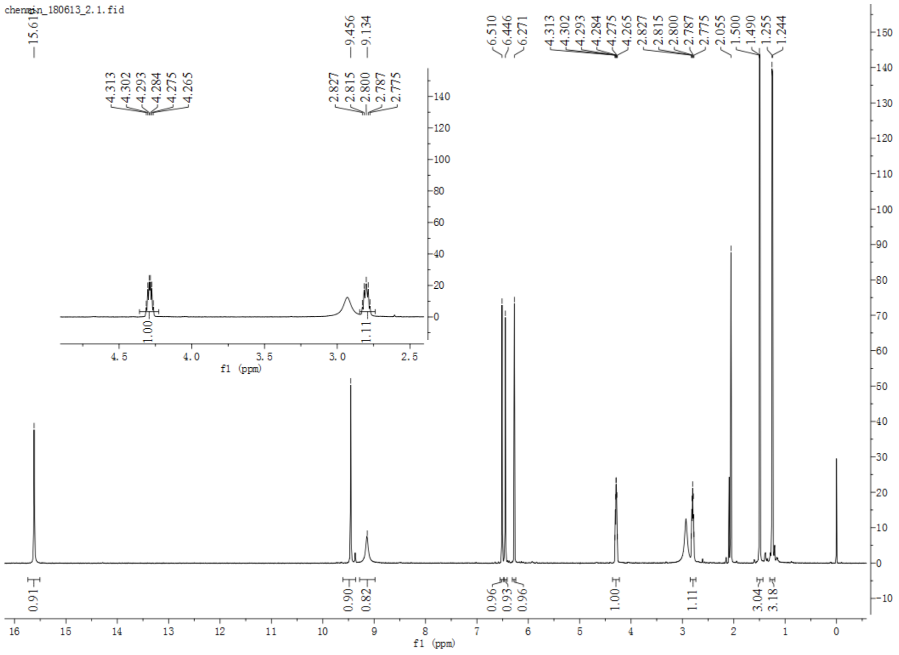

Figure S8. ^{1}H NMR spectrum of compound **2** in acetone-d_6 (600 MHz).

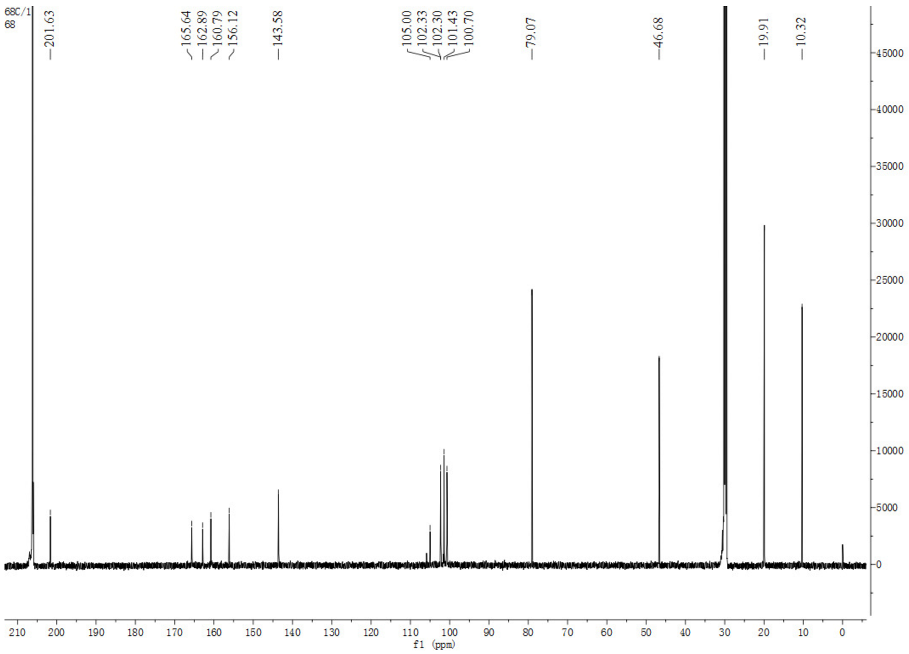

Figure S9. ^{13}C NMR spectrum of compound **2** in acetone-d_6 (150 MHz).

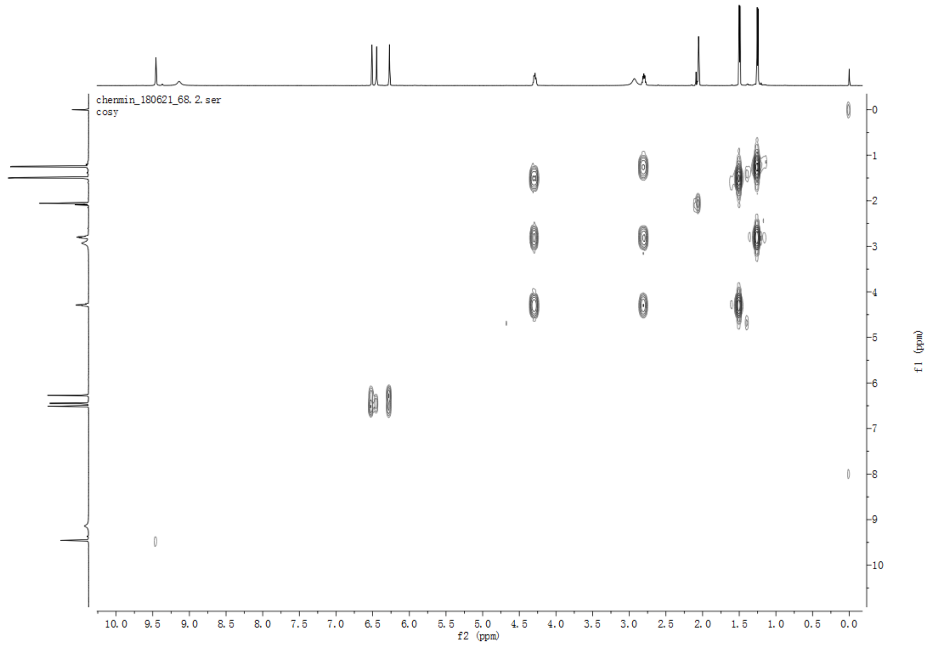

Figure S10. ^{1}H-^{1}H COSY spectrum of compound **2** in acetone-d_6 (600 MHz).

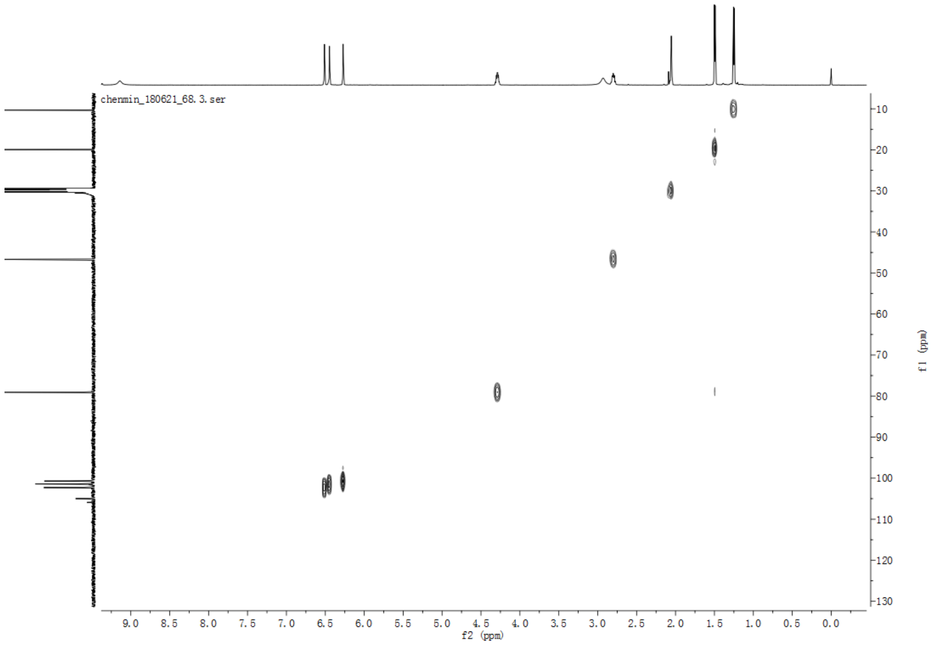

Figure S11. HSQC spectrum of compound **2** in acetone-d_6 (600 MHz).

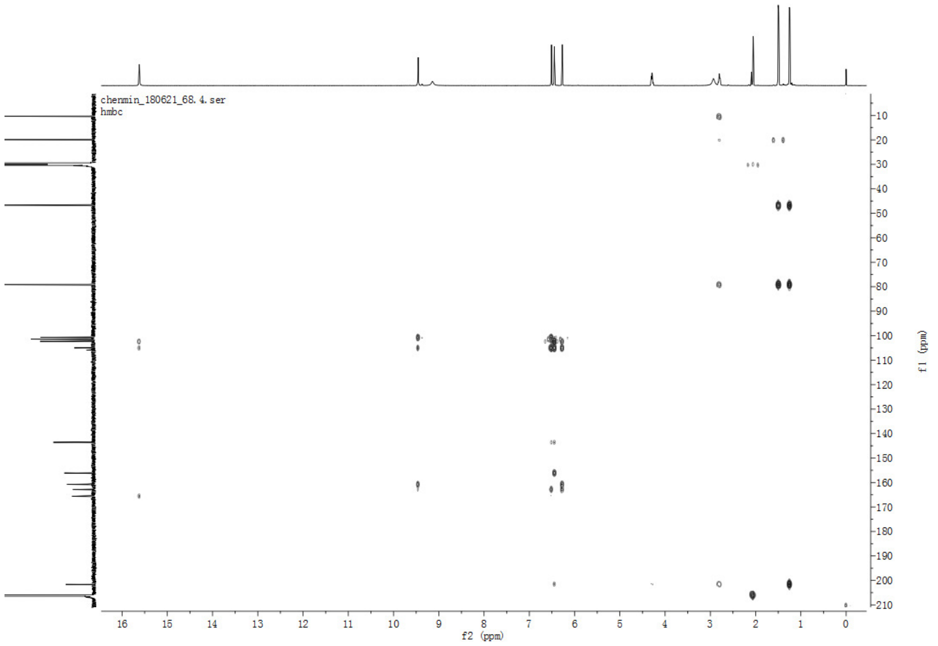

Figure S12. HMBC spectrum of compound **2** in acetone-d_6 (600 MHz).

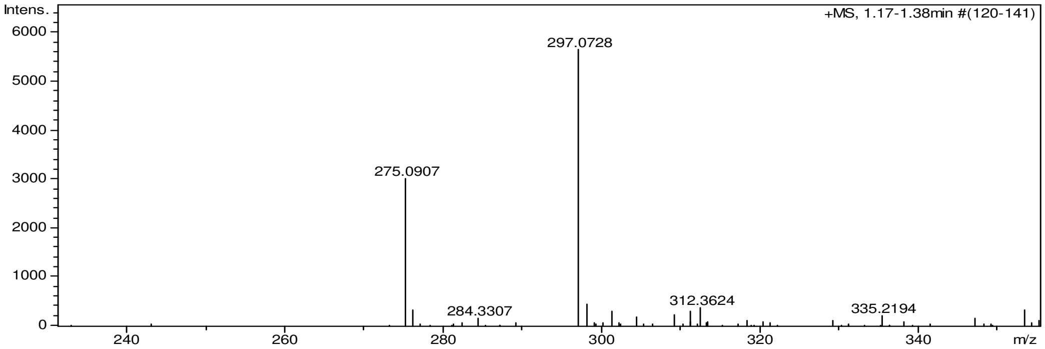

HRESIMS *m/z* 275.0907 (calcd for $C_{15}H_{15}O_5$, 275.0914),

297.0728 (calcd for $C_{15}H_{14}NaO_5$, 297.0733)

Figure S13. HRESIMS spectrum (positive ion mode) of compound **2**.

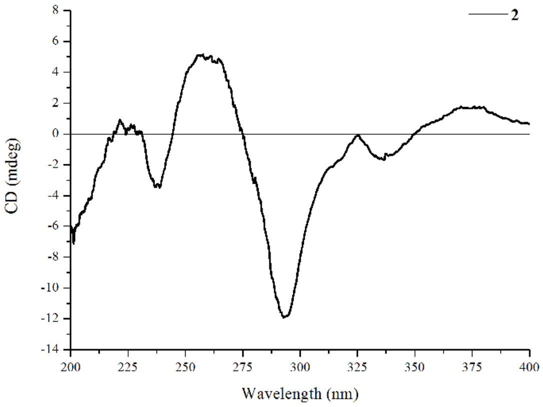

Figure S14. ECD spectrum of compound **2**.

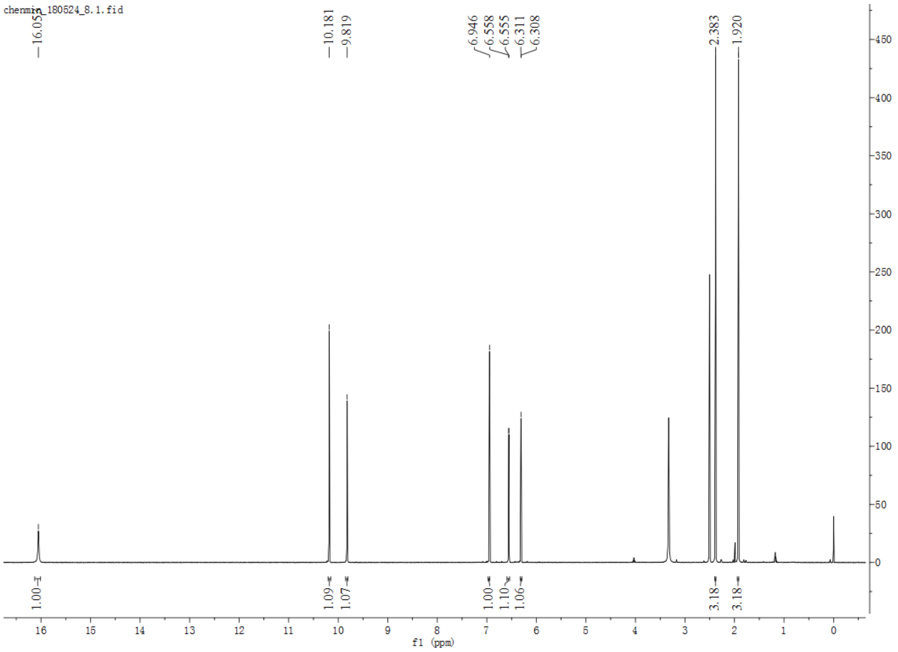

Figure S15. ^{1}H NMR spectrum of compound **3** in acetone-d_6 (600 MHz).

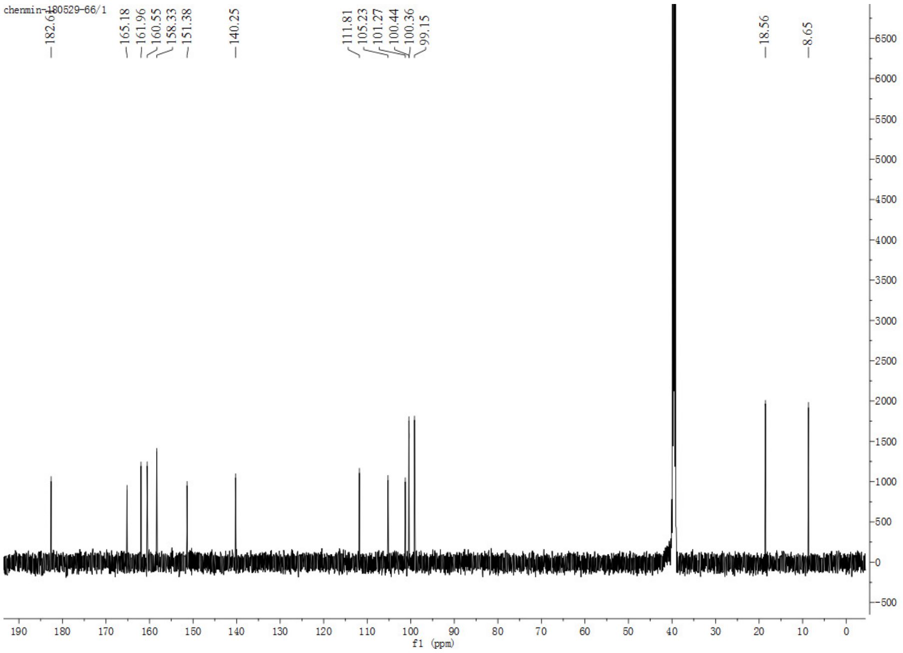

Figure S16. ^{13}C NMR spectrum of compound **3** in acetone-d_6 (150 MHz).

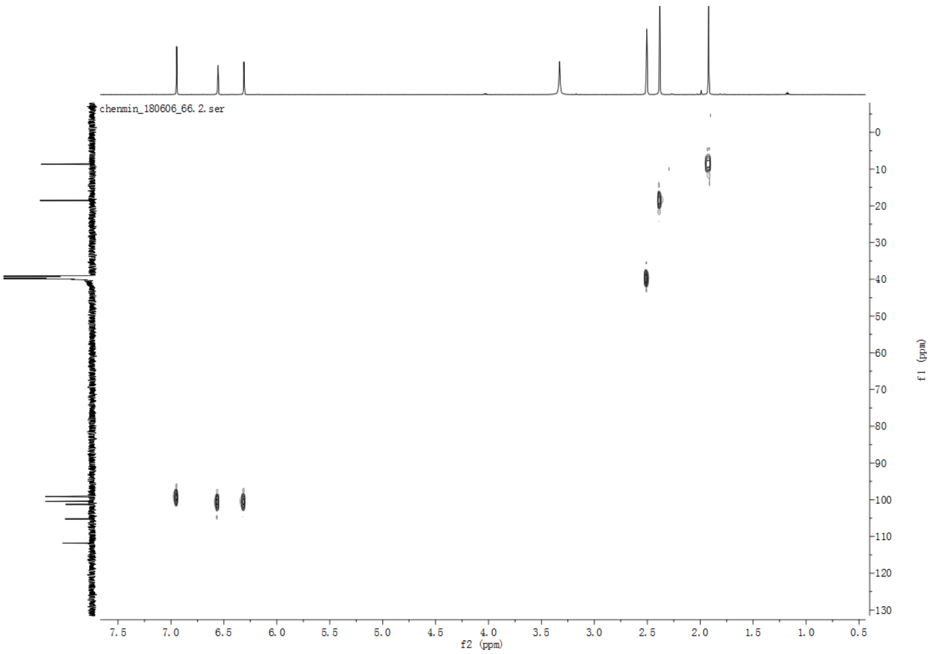

Figure S17. HSQC spectrum of compound **3** in acetone-d_6 (600 MHz).

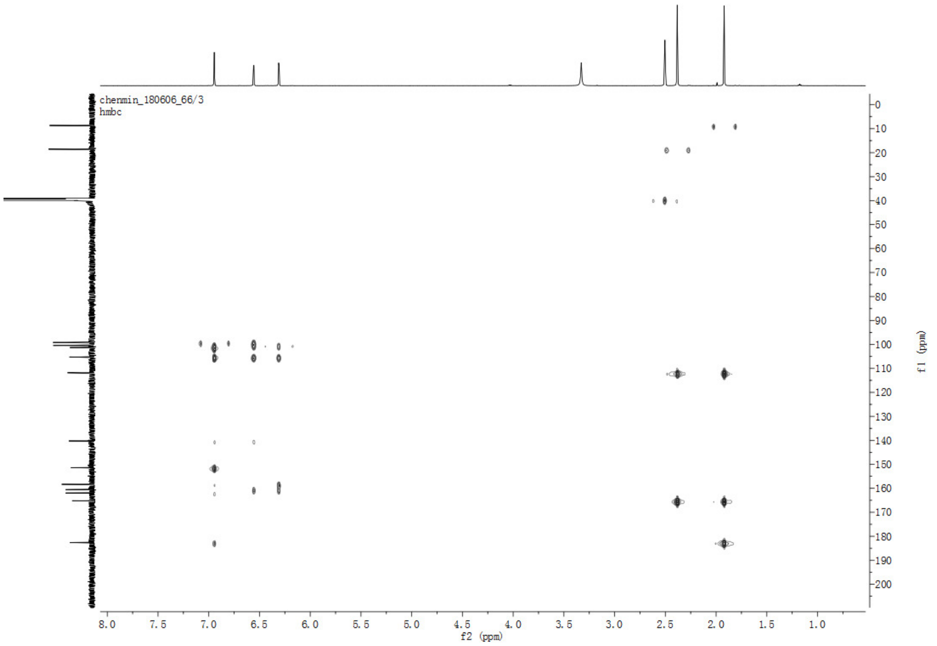

Figure S18. HMBC spectrum of compound **3** in acetone-d_6 (600 MHz).

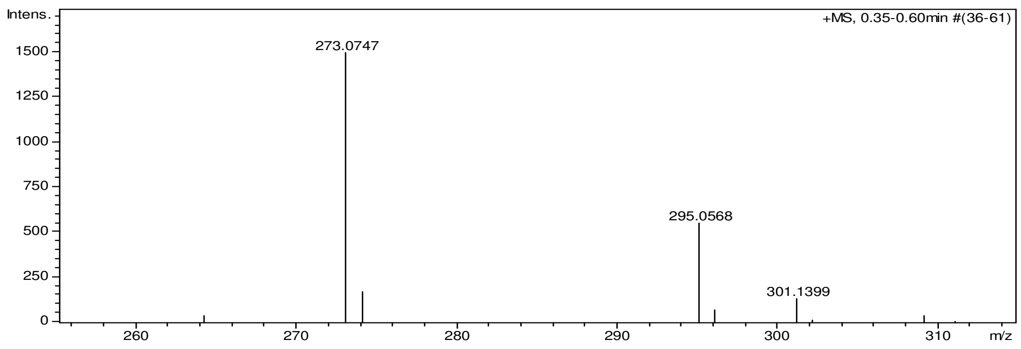

HRESIMS *m/z* 273.0747 (calcd for $C_{15}H_{13}O_5$, 273.0757),

295.0568 (calcd for $C_{15}H_{12}NaO_5$, 295.0577)

Figure S19. HRESIMS spectrum (positive ion mode) of compound **3**.

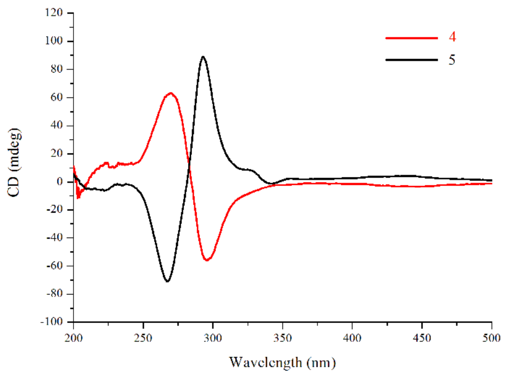

Figure S20. ECD spectrum of compounds **4** and **5**.

The ^{1}H NMR, ESIMS and ECD data of **4** and **5.**

Isochaetochromin B$_1$ (4): yellow amorphous powder; ECD (0.30 mM, MeOH) λ_{max} ($\Delta\varepsilon$) 295 (−55.52), 270 (+62.20) nm; ^{1}H NMR (acetone-d_6, 600 MHz) δ_H 6.71 (1H, s, H-10), 6.55 (1H, s, H-7), 6.43 (1H, s, H-7′), 6.13 (1H, s, H-10′), 4.71 (1H, dq, J = 3.0, 5.4 Hz, H-2), 4.23 (1H, dq, J = 13.2, 5.4 Hz, H-2′), 2.85 (1H, dq, J = 3.0, 6.6 Hz, H-3), 2.77 (1H, dq, J = 13.2, 6.6 Hz, H-3′), 1.41 (3H, d, J = 5.4 Hz, CH_3-11), 1.41 (3H, d, J = 5.4 Hz, CH_3-11′), 1.22 (3H, d, J = 6.6 Hz, CH_3-12), 1.22 (3H, d, J = 6.6 Hz, CH_3-12′). ESIMS *m/z* 547 [M + H]$^+$.

Isochaetochromin B_2 (5): yellow amorphous powder; ECD (0.30 mM, MeOH) λ_{max} ($\Delta\varepsilon$) 294 (+88.07), 267 (−70.79) nm; ^{1}H NMR (acetone-d_6, 600 MHz) δ_H 6.71 (1H, s, H-10), 6.56 (1H, s, H-7), 6.44 (1H, s, H-7′), 6.13 (1H, s, H-10′), 4.72 (1H, dq, J = 3.0, 5.4 Hz, H-2), 4.24 (1H, dq, J = 11.4, 5.4 Hz, H-2′), 2.85 (1H, dq, J = 3.0, 6.6 Hz, H-3), 2.77 (1H, dq, J = 11.4, 6.6 Hz, H-3′), 1.41 (3H, d, J = 5.4 Hz, CH_3-11), 1.41 (3H, d, J = 5.4 Hz, CH_3-11′), 1.23 (3H, d, J = 6.6 Hz, CH_3-12), 1.23 (3H, d, J = 6.6 Hz, CH_3-12′). ESIMS m/z 547 $[M + H]^+$.

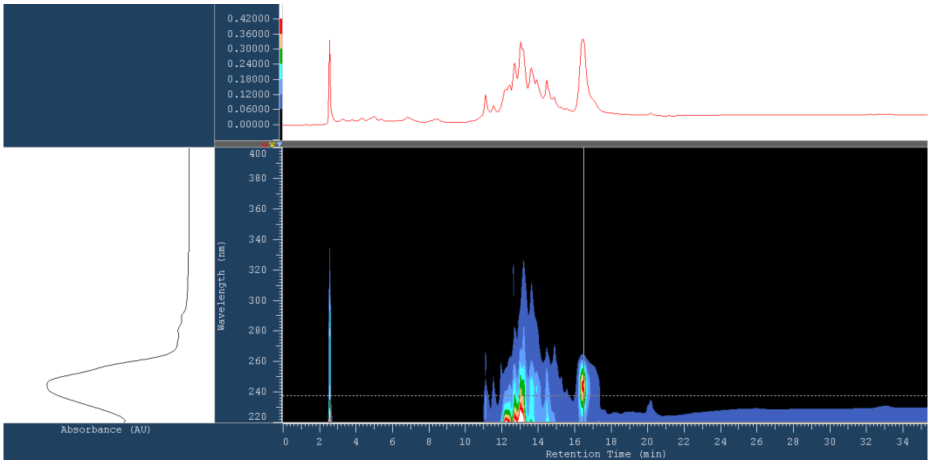

Figure S21. HPLC chromatogram of the crude extract from the fungus *Penicillium* sp. HK1-22.

HPLC was performed on a Hitachi L-2000 system using an analytical C_{18} (Apollo, 5 μm, 250 mm × 4.5mm) column coupled with a 2455 UV detector. The conditions of the HPLC analysis: solvents: A, water; B, MeOH. Linear gradient: 0 min, 20% B; 5 min, 20% B; 10 min, 80% B; 15 min 100% B; 60 min 100%. Temperature: 30 ºC. Flow rate: 1 mL/min. UV detection at λ = 220 nm.
